# Effects of Brook Trout Invasion on Behavioral and Dietary Shifts in Brown Trout

**DOI:** 10.1002/ece3.70995

**Published:** 2025-03-20

**Authors:** B. Austad, L. Závorka, J. Cucherousset, J. Höjesjö

**Affiliations:** ^1^ Department of Biological and Environmental Sciences University of Gothenburg Gothenburg Sweden; ^2^ WasserCluster Lunz–Biologische Station Inter‐University Center for Aquatic Ecosystem Research Lunz am See Austria; ^3^ Centre de Recherche sur la Biodiversité et l'Environnement (CRBE) Université de Toulouse, CNRS, IRD, Toulouse INP, Université Toulouse 3 – Paul Sabatier (UT3) Toulouse France

**Keywords:** animal personality, behavioral ecology, brook trout, brown trout, invasive species, niche shift, trophic niche

## Abstract

Behavioral variation within a population is generally maintained by frequency dependent selection, allowing various personalities to coexist. Bolder individuals usually engage in more risky behaviors that can gain fitness benefits such as growth under certain conditions. Therefore, it has been suggested that there should be a link between personality and dietary niches, but the results so far are inconsistent. In addition, the equilibrium of the distribution of behavioral traits and the trophic niche of native populations may shift following the introduction of an invasive species. Here, using the invasive brook trout ( *Salvelinus fontinalis* ) and native brown trout ( *Salmo trutta* ) as model species in two different natural streams in Sweden, we aimed to test whether (1) the trophic niche of native brown trout living in allopatry and brown trout living in sympatry with brook trout differ and (2) bolder brown trout individuals utilize a different foraging niche. Our results suggest that there is a dietary niche convergence between brown trout and brook trout, which likely is a result of brook trout invasion, but that the trophic niche of native brown trout varies across streams, possibly due to differing invasion impacts (varying ratio of brook trout to brown trout). We also found a strong positive correlation between trophic position and personality of brown trout irrespective of the presence of brook trout.

## Graphical abstract

This study investigated the effects of invasive brook trout on the trophic niche of native brown trout in two natural streams in Sweden. Results showed dietary niche convergence between the species, likely due to brook trout invasion, but with variation across streams. Additionally, a strong positive correlation between the trophic position and boldness of brown trout was observed, regardless of brook trout presence.

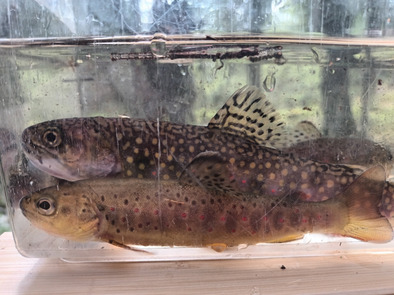

## 1 Introduction

Within a population of animals, individuals display different behaviors that persist temporally with a heritable component, often defined as personality or, if several behavioral traits covary across time and contexts, behavioral syndromes (Toscano et al. 2016; Stamps and Groothuis 2010; White et al. 2013). This within‐population variation is maintained due to ecological factors, which may allow diverse traits to persist within a population (Wolf et al. 2012). Therefore, disruption of this population equilibrium caused by anthropogenic‐induced environmental changes, such as the introduction of non‐native species, can have unforeseen effects on the distribution of behavioral traits (Závorka et al. 2017).

An important aspect of animal behavior is boldness, typically defined as an individual's tendency to engage in risky behaviors. This trait is also connected to other characteristics, as outlined in the pace‐of‐life behavioral syndrome hypothesis, which describes a continuum of related behaviors. This predicts that individuals on the “slow” end of this continuum would be shy, likely to avoid risky situations and display less aggressive behavior compared to “fast” individuals (Royauté et al. 2018; Adriaenssens and Johnsson 2010; Sloan Wilson et al. 1994). The fitness consequences of boldness are suggested to depend on a growth–mortality trade‐off, both driven by the intensity of predation pressure and by the growth benefits that bold individuals can potentially gain from engaging in risky behavior (Mittelbach et al. 2014).

A key outcome of boldness and other personality traits is their realized ecological effects, such as the linkage between boldness and trophic niche. Trophic niche can change quickly and is influenced by resource availability and trophic interactions, as well as the behavioral, physiological, and genetic characteristics of individuals (Moosmann et al. 2021). Mittelbach et al. (2014) suggested that fish personality could influence ontogenetic dietary shifts. Furthermore, a study on sticklebacks ( *Gasterosteus aculeatus* ) found a positive correlation between latency to explore and increased foraging on Chironomidae larvae in the littoral zone, although this increase was also related to sex of the fish (Theódórsson and Ólafsdóttir 2022). On the other hand, Kerr and Ingram (2020) did not find that personality was associated with individual trophic niche for common bully ( *Gobiomorphus cotidianus* ). Therefore, there is a need to further explore the circumstances in which the association between boldness and trophic niche may occur.

**FIGURE 1:**
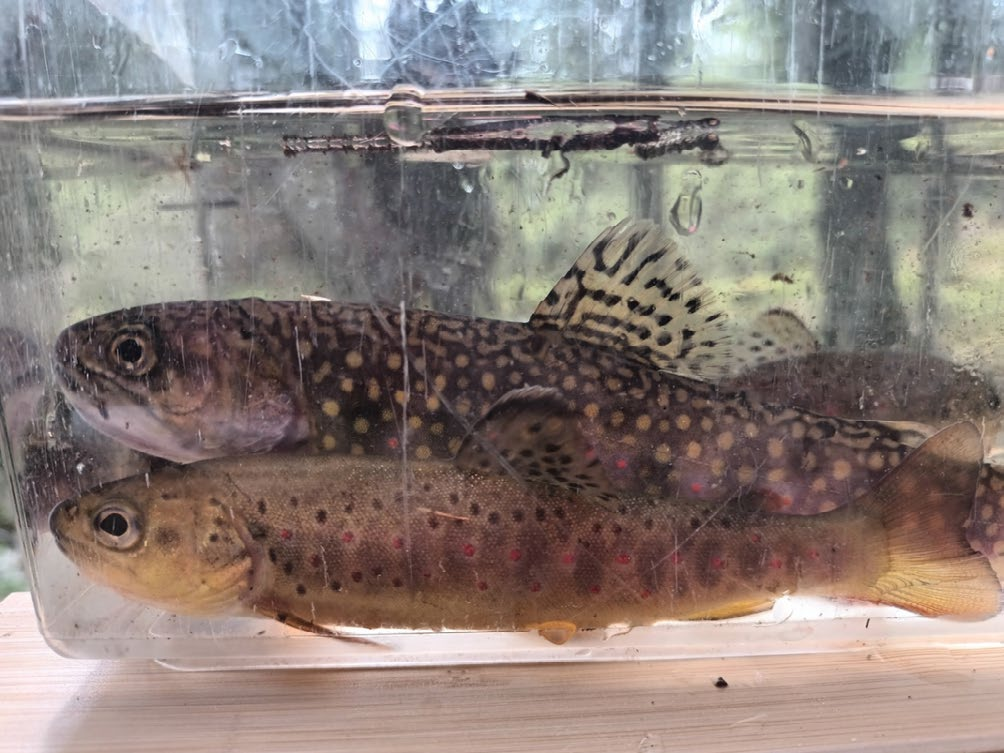
A brook trout (above) and a brown trout (below), both captured in the same stream section.

Among the previously mentioned anthropogenic effects is the issue of invasive species, which are commonly defined as introduced species whose introduction and/or spread affect the ecosystem either directly or indirectly to the detriment of local species (Francis and Chadwik 2012; Weis and Sol 2016; NOBANIS n.d.). An interesting case in this context is that of brook trout ( *Salvelinus fontinalis* ) and brown trout ( *Salmo trutta* ) (Figure 1). These are salmonids originating from North America and Eurasia, respectively (Jonsson and Jonsson 2011). In Sweden, brook trout are considered an invasive alien species. They have been released since 1892, with a peak between 1950 and 1960, resulting in approximately 300 self‐reproducing populations today (Havs och vattenmyndigheten 2020).

Brook and brown trout tend to displace each other along a longitudinal gradient due to differences in habitat preferences (Kirk et al. 2018). In sympatric habitats, the direction of asymmetric competition between the species may be context‐dependent. Horká et al. (2017) determined brown trout to be the stronger competitor in sympatric localities based on an observed diet divergence driven by brook trout changing their diet but not brown trout. On the other hand, Lovén Wallerius et al. 2022 demonstrate asymmetric competition in the opposite direction at certain life stages; brook trout have a competitive advantage over brown trout due to their earlier emergence and larger body size. In some streams, this has led to a distribution of brown trout in allopatry at the lower end of the stream, and brook trout either in allopatry or in sympatry with brown trout at the upper part of the stream (Korsu et al. 2007; Závorka et al. 2017). Previous studies have found that brown trout living in sympatry with brook trout display changes in several key phenotypic and ecological niche traits not connected to the specific stream gradient (Závorka et al. 2017; Jonsson and Jonsson 2011). For example, sympatric brown trout with brook trout have been shown to consume more terrestrial prey than allopatric conspecifics (Cucherousset et al. 2007), have different head morphology (Závorka et al. 2020), be more diurnal (Larranaga et al. 2019), have smaller home ranges (Závorka et al. 2017), and a higher tendency to aggregate with other individuals (Lovén Wallerius et al. 2017). However, it remains unclear how these changes relate to important behavioral traits such as boldness.

Brown trout is regarded as a generalist stream‐dwelling salmonid, and they are considered to be drift feeders (Sánchez‐Hernández et al. 2012; Sánchez‐Hernández and Cobo 2013). They consume terrestrial and benthic invertebrates and sometimes smaller fish or eggs available in the stream. They display a great deal of flexibility, adapting as they grow and as seasons change (e.g., through relying more on terrestrial prey during summer when aquatic biomass is low) (Sánchez‐Hernández 2024). Nakano, Fausch, and Kitano (1999) describe this flexibility in native Dolly Varden charr; when drifting prey is scarce, charr shift foraging modes to benthos feeding. Further, Nakano, Kawaguchi, et al. 1999 show that invading rainbow trout actively select terrestrial prey over aquatic prey (due to the larger size) and consume the majority of the total input of terrestrial prey into a stream reach during summer, but if the input of terrestrial prey is reduced, rainbow trout will forage more intensively on aquatic invertebrates. So, while it initially may seem that salmonids simply feed on prey items proportional to availability in the stream in which they reside, this is not necessarily entirely true, as they do not always consume the most abundant and/or profitable taxa (Sánchez‐Hernández and Cobo 2013). Because although resource availability is foundational, predators may adjust their niches not only based on prey availability but also in response to or as a result of other factors like ontogeny and competition (Sánchez‐Hernández et al. 2021). Additionally, personality traits can influence dietary choices; for example, consuming a higher proportion of benthic aquatic prey could require a more mobile and active modus operandi typical of a bolder fish (Hart 1997). Furthermore, consumption of aquatic prey has been found to be positively correlated with growth rate (Evangelista et al. 2014), which could also be a result of a bold personality where growth is gained in a trade‐off with mortality.

The aim of the current study is to examine how the native brown trout's dietary niche may be affected by the presence of the invasive brook trout, and how differences in boldness are linked to trophic niche. In this study, we used stable isotope analyses to examine whether utilized prey items differ between brown trout living in allopatry and brown trout living in sympatry with brook trout of separate stretches of two different streams in the south of Sweden. We also assessed boldness in the field to evaluate to what extent bold individuals utilize a different foraging niche compared to their shy conspecifics.

## 2 Methods

### 2.1 Study Streams and Fish

**FIGURE 2:**
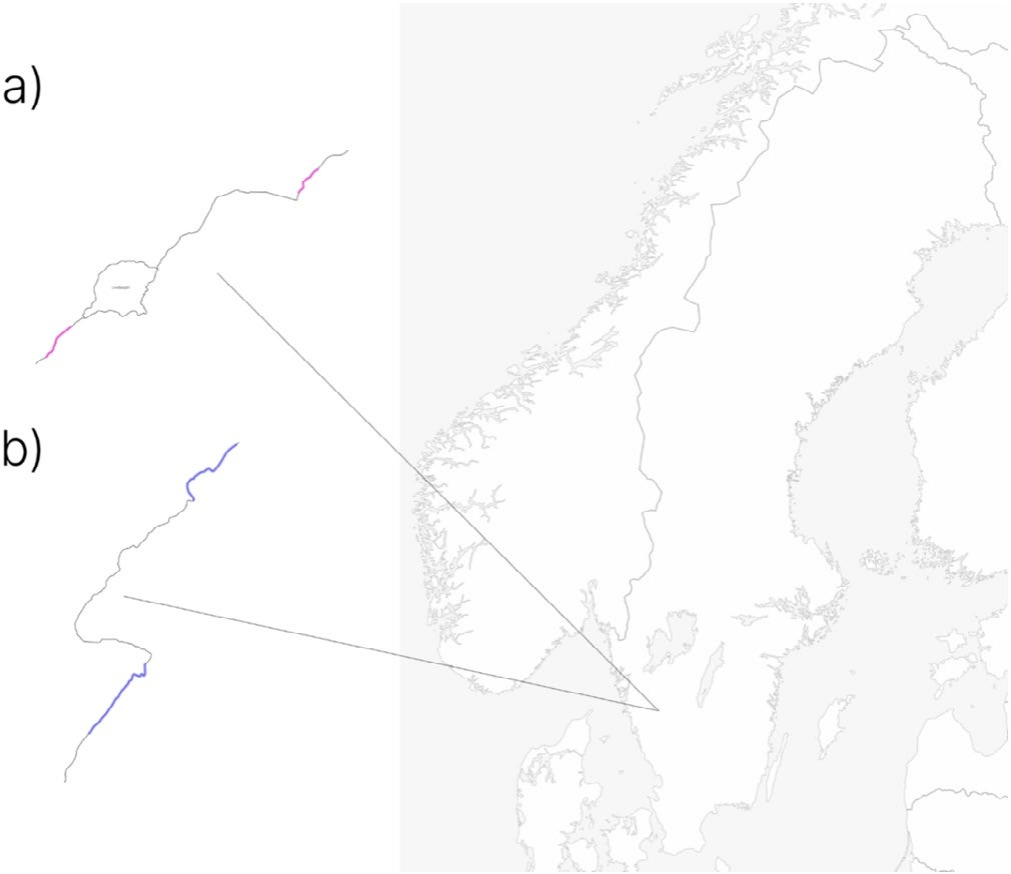
Map showing the streams (a) Lindåsabäcken sampling sites and (b) Ringsbäcken sampling sites.

The study was conducted in two natural streams: Lindåsabäcken and Ringsbäcken, both located in the Viskan catchment area near Borås, Sweden. For both streams, an allopatric section inhabited by brown trout only downstream and a sympatric section inhabited by both brook trout and brown trout further upstream were used as the fishing stretches (Figure 2). Fish were sampled in August 2021 using electrofishing (Smith‐Root LR‐20B, Vancouver, Washington, USA) (Appendix A1).

### 2.2 Boldness Scoring

**FIGURE 3:**
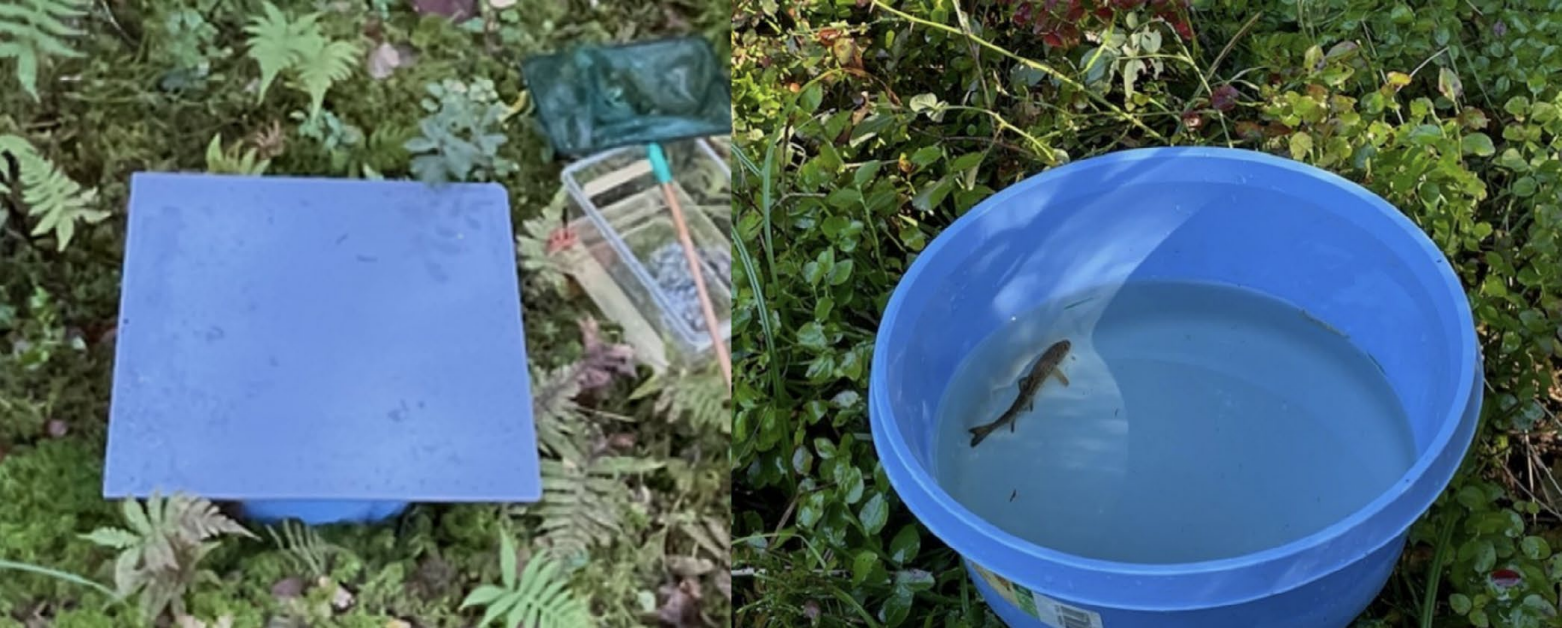
The in‐field boldness scoring setup. Fish were carefully netted from the stream into a prefilled round container with opaque sides that was immediately covered with an opaque cover for 120 s before it was swiftly removed and the movements of the fish were observed and timed by a live coder.

After being captured, all fish were placed in in‐stream boxes for 24 h to recover from electrofishing before being scored for boldness in the field using a modified open field test. In this test, latency to start swimming when exposed to a novel environment was used to score boldness (see McGlade et al. 2022). Scoring fish in the field did not allow for a standardization of light and weather conditions (although the scoring was performed at a similar time of day and year for all the fish) nor did it allow for scoring each fish more than once, but this approach reduces stress in the fish from handling, transport, and laboratory captivity, as well as minimizing the time fish spent out of the stream, allowing for an assessment of boldness that would be as close as possible to the behavior displayed in the stream, as well as minimizing the time in which the fish were in distress (Závorka et al. 2020). Each fish was individually placed into a small round container (30 cm in diameter) with opaque sides, filled to a 10 cm depth with water collected directly from the stream. The containers were immediately covered with an opaque plastic lid to block out all light (Figure 3). The fish were kept in the box for 120 s to acclimatize before the cover was swiftly removed and a timer started. The trial for each fish lasted 300 s, and we recorded whether the fish moved—1 or stayed still—0 during this period. We assumed that bold fish were more likely to move during the test, as we expect bolder fish to display a shorter latency to start swimming in the novel environment (see for example Theódórsson and Ólafsdóttir 2022). Movement was defined as fish moving at least half its body length away from the starting point.

### 2.3 Stable Isotope Analysis (SIA)

A small clip of each fish's pelvic fin was collected. Samples of putative aquatic prey were collected from three kick‐samples at three random spots in each section and terrestrial prey was collected using hand nets at three spots near the stream. A subsample of size matched fish (Appendix A1) was used for stable isotope analysis of carbon (δ^13^C) and nitrogen (δ^15^N) (Appendix A2). Carbon and nitrogen stable isotopes can be used to understand and examine trophic relationships and energy flows within an ecosystem using ratios of the isotopes between consumers and their diets. Carbon mainly provides information about the origin of energy sources because it marginally increases from diet to consumer while nitrogen will increase with each trophic transfer allowing for estimation of trophic positions of the consumer (Logan et al. 2008; O'Reilly et al. 2002). Fin clips and prey items were rinsed with distilled water, kept on ice, brought into the laboratory, and dried at 60°C for 48 h (see prey sample list in Appendix A3). Subsequently, they were manually grinded into a homogenous powder, weighed, and poured into tin cups. Samples were analyzed at the ISOGOT facility at the Department of Earth Sciences, University of Gothenburg, Sweden. Variation in lipid content of the samples may constitute biases of the isotope analyses because lipids are depleted in δ^13^C (Post et al. 2007). The fish samples included in this study were generally low in lipid content, so no correction for lipids was deemed necessary (mean ± SD; brown trout: 3.4 ± 0.09, brook trout: 3.2 ± 0.1).

### 2.4 Statistical Analyses

Statistical analyses and figures were produced using R version 4.4.0 (R Core Team 2023).

The isotopic niche size for each group and isotopic niche overlap between brook trout and brown trout in the sympatric sections were analyzed using the Stable Isotope Bayesian Ellipses (SIBER) in R (Jackson et al. 2011). The standard ellipse area (SEA) was used as a measure of stable isotope niche size and can also be used to determine isotopic niche overlap between consumer species. Due to sample sizes, standard ellipse area corrected for sample size (SEAc) was calculated, which includes 40% of the stable isotope variability, thereby representing the core isotopic niche. Isotopic values were corrected to account for variability in resources between the sites following Olsson et al. (2009):

$$\delta^{13}C\;\text{corri}=\delta^{13}C\;i-\delta^{13}C\;\mathrm{inv}/\text{CRinv}$$

where *δ* ^13^C_corr*i*_ is the corrected carbon isotopic ratio for individual *i*, *δ* ^13^C_*i*_ is the carbon isotopic ratio for individual *i*, *δ* ^13^C_inv_ is the average carbon isotope ratio of the invertebrates and CR_inv_ is the carbon range (*δ* ^13^C_max_ − *δ* ^13^C_min_). The correction following the same equation was done for δ^15^N (Fedorčák et al. 2025). The isotopic values corrected for the prey baseline were then used in all models. Therefore, in the results unless stated otherwise all outputs are based on baseline corrected isotopic values.

To test for differences in isotopic niches, we conducted two sets of analyses. First, we examined differences between allopatric and sympatric brown trout, and between sympatric brown and brook trout in each stream using multivariate analysis of variance (MANOVA). For each stream, we constructed models with corrected δ^13^C and δ^15^N as response variables and species (brown trout or brook trout) as the fixed effect, including fork length as a covariate to account for size‐related differences in isotopic values. When MANOVA indicated significant differences in the overall isotopic niche, we proceeded with univariate ANOVAs for each isotope separately to determine the specific nature and direction of these differences.

Second, to examine how individual isotopic niches influenced boldness, we analyzed behavioral responses using two approaches: (1) a generalized linear model with a binary response (whether an individual moved within 300 s), and (2) a linear model of the time until first movement for those individuals that did move. Both models included corrected δ^13^C, δ^15^N, fork length, stream of origin, and sympatry with brook trout as explanatory variables. We analyzed brown and brook trout separately, excluding the sympatry variable for brook trout models.

## 3 Results

**FIGURE 4:**
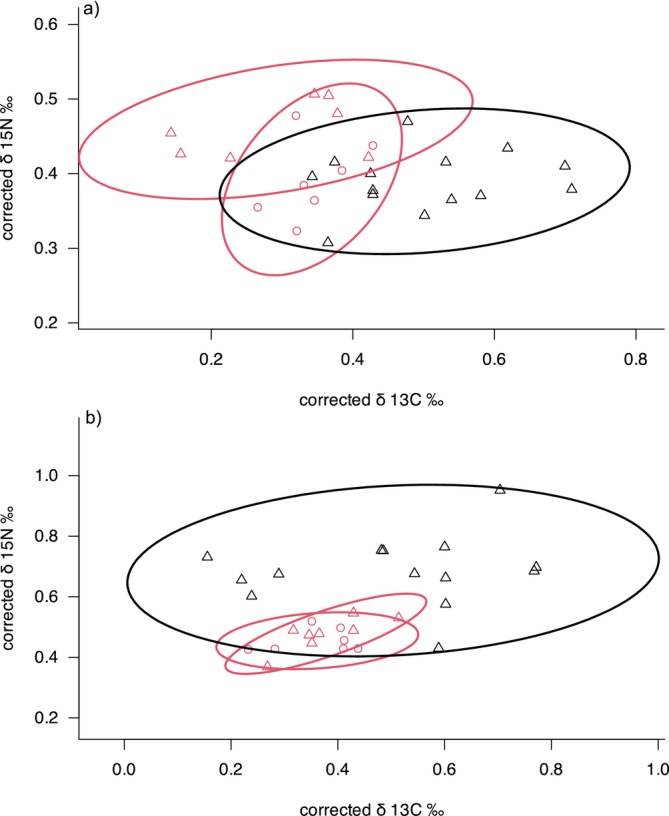
Stable isotope niches (SEA) between brook trout (circles) and brown (triangles) trout in the sympatric and allopatric stretches of (a) Lindåsabäcken (b) Ringsbäcken. Sympatric and allopatric stretches in each stream are indicated by red and black symbols, respectively.

Overlap in stable isotope niche between brook trout and brown trout was approximately 52% in Ringsbäcken, which was larger than the 28% overlap found in Lindåsabäcken (Figure 4, Appendix A2). Overlap in stable isotope niche of allopatric and sympatric brown trout was 9% in Ringsbäcken and 17% in Lindåsabäcken (Figure 4). The broadest isotopic niche size was found in brown trout of Ringsbäcken allopatry (SEAc = 0.079), which differed from Ringsbäcken sympatry where brook trout and brown trout had similar isotopic niche sizes (SEAc = 0.011 and 0.009, respectively). In Lindåsabäcken, the standard ellipse areas were similar for brown trout in allopatry and sympatry (SEAc = 0.015 and 0.016, respectively). For brook trout in Lindåsabäcken, the stable isotope niche size was similar to the niche size of brook trout in Ringsbäcken (SEAc = 0.009).

There was a significant shift in the mean isotopic niche of allopatric and sympatric brown trout in both observed streams (Ringsbäcken: Manova, *F*_2;18_ = 19.29, *p* < 0.001 and Lindåsabäcken: Manova, *F*_2;15_ = 20.91, p < 0.001). In Lindåsabäcken, this shift was driven by an increase of δ^15^N (*F*_1;16_ = 30.47, p < 0.001) and a decrease of δ^13^C (*F*_1;19_ = 5.55, *p* = 0.03; Figure 4a) in sympatry with brook trout compared to allopatry. This result indicated a higher reliance of sympatric brown trout on aquatic rather than terrestrial prey compared to the allopatric conspecifics because the aquatic prey has typically more depleted δ^13^C compared to the terrestrial prey. In Ringsbäcken, this shift was driven by a decrease of δ^15^N in sympatry with brook trout (*F*_1;19_ = 22.1, *p* < 0.001), but no significant change in δ^13^C (*F*_1;19_ = 3.62, *p* = 0.07; Figure 4b). The mean isotopic niche of sympatric brook and brown trout differed in Lindåsabäcken (MANOVA, *F*_2;10_ = 7.22, *p* = 0.011), as sympatric brown trout had a higher δ^15^N (*F*_1;11_ = 13.28, *p* = 0.004), but similar δ^13^C (*F*_1;11_ = 0.72, *p* = 0.413) as brook trout (Figure 4a). The mean stable isotope niche of sympatric brook and brown trout did not significantly differ in Ringsbäcken (MANOVA, *F*_2;11_ = 1.04, *p* = 0.387; Figure 4b).

**FIGURE 5:**
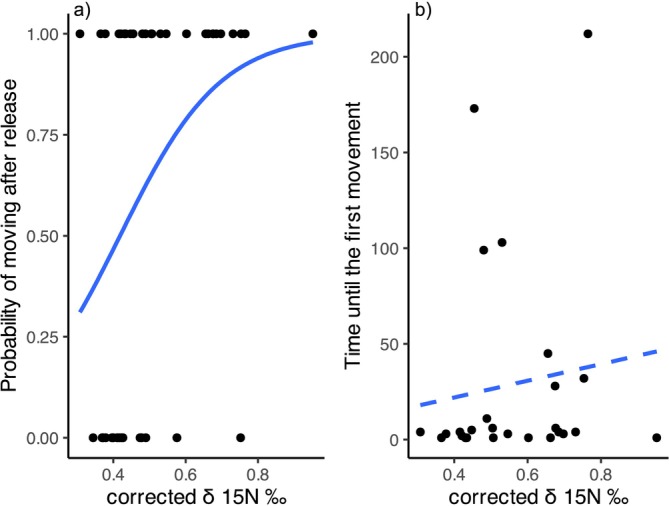
Relationship between nitrogen stable isotope values (δ^15^N corrected) and the probability of brown trout moving within 5 min after the release to the test box (a) and the time in seconds until the first movement among those brown trout individuals that did move (b). Blue line indicates the main trend of the relationship between the variables, solid and dashed lines indicate statically significant and non‐significant results, respectively.

We found that the probability of brown trout moving within 300 s increased significantly with increasing value of δ^15^N (*F*_1;39_ = 6.24, *p* = 0.0169; Figure 5). No other tested variable (δ^13^C, fork length, stream of origin, sympatry with brook trout) had a significant effect on the behavioral response of brown trout (summary in Table 1). When we analyzed the time until the first movement in the subset of individuals that did move within the 300 s interval, we found no significant effect of δ^15^N (Figure 5b) or any other explanatory variable. The behavioral response of brook trout was not significantly related to any explanatory variable considered, regardless of whether we used the probability to move or time until the first movement as the response variable.

**TABLE 1:** Results from generalized linear model (binomial distribution with logit link) testing the effects of isotopic and morphological variables on the probability of brown trout moving within the set time interval.

Variable	Estimate	95% CI (Lower)	95% CI (Upper)	*F*‐statistic	*p*
δ^15^N	0.45	0.1	0.8	6.24	0.0169*
δ^13^C	0.12	−0.15	0.39	0.45	0.505
Fork length	−0.05	−0.2	0.1	0.12	0.73
Stream	0.08	−0.1	0.26	0.3	0.587
Treatment (Sympatry)	−0.02	−0.18	0.14	0.02	0.88

*denotes significance of *p* ≤ 0.05.

## 4 Discussion

As expected, the niche overlap between native brown trout and invasive brook trout was greater in sympatry than in allopatry when adjusted for prey baselines in both streams tested (Figure 3). In Ringsbäcken, interspecific competition appeared to restrict the isotopic niche of brown trout, resulting in a narrower niche compared to allopatric populations. This finding supports the niche variation hypothesis (NVH), which suggests that populations with narrower niches exhibit less variation between individuals and fewer dietary specialists (Bolnick et al. 2007, 2010; Sánchez‐Hernández et al. 2021). For example, Baker et al. (2022) found that brook trout populations with broader population‐level niches contained more individual specialists. The introduction of brook trout likely increases the competition for prey sources, which may selectively impact brown trout with certain behavioral traits (Wauters et al. 2019). The observed narrowing of the brown trout's isotopic niche in our study suggests that competition with brook trout may be driving the loss of dietary specialists, as brown trout are forced to adjust their diet to a more limited range of prey.

A curious aspect of the current study is that it did not find the same pattern of increased terrestrial prey consumption as previously described (Závorka et al. 2017; Cucherousset et al. 2020). This is particularly noteworthy as Závorka et al. (2017) conducted their study in the same stream and approximately the same stream section. A potentially important difference was, however, the season in which the studies were conducted. Závorka et al. conducted their study during spring and early summer, while the current study took place during late summer. Sánchez‐Hernández et al. (2021) posit that prey diversity and abundance are higher during early summer, and that when food is not limited, prey diversity rather than competition drives niche variation. This seasonal variation could mean that during late summer, when resources are more limited, competition becomes the primary driver of niche variation rather than prey diversity promoting broader dietary shifts.

On the other hand, novel competition pressure from brook trout may cause brown trout to shift their isotopic niche without narrowing it (i.e., as we observed in the second stream in the present study). Previous studies have shown brook trout to often induce dietary shifts toward more terrestrial prey in sympatric brown trout (but see Horká et al. 2017) populations compared to allopatric populations; this diet shift represents a niche convergence with brook trout that also feeds predominantly on terrestrial prey (Cucherousset et al. 2020; Závorka et al. 2017, 2021). However, it is worth noting that our study indicates an opposite pattern to these previous works, since we found a shift toward more aquatic prey (in Lindåsabäcken) or no shift of the mean trophic niche of brown trout (in Ringsbäcken) in sympatry with the brook trout. This is an important finding as it suggests that the changes in the trophic niche of brown trout competing with an invasive brook trout are more context dependent than previously thought, which again aligns with the broader patterns of ontogenetic and seasonal dietary shifts observed in salmonids, where environmental and competitive pressures can lead to significant changes in feeding behavior, emphasizing the subjective nature of these shifts (Sánchez‐Hernández 2024).

Although the two streams used in this study are very similar both in size, location, and in the general features of the stream (discharge, shading etc.), the sympatric stretch of Lindåsabäcken sympatry has a higher proportion of brook trout then Ringsbäcken (80 vs. 50%, ratios based on separate electrofishing sampling when all fish in the stretch that were observed were counted regardless of size and species). Therefore, streams differ in intensity of invasion and propagule pressure on the native community. When native and invasive species occupy the same trophic level, competition is the primary driver of the invader's impact; this competition can be density‐dependent, potentially causing non‐linear declines in population sizes (Bradley et al. 2019). Perhaps the brown trout in Lindåsabäcken sympatry are displaced from their original niche by brook trout to the point where surviving individuals have been forced to adopt a different foraging strategy than brook trout to avoid the competition for prey.

In this study, each fish's boldness was assessed using a single measurement. Research across various taxa has shown that boldness tends to remain robust over time, indicating that a single measurement can often reliably capture an individual's behavioral profile (Carlson et al. 2024; Dingemanse and Wolf 2010). This consistency supports the validity of using one‐time assessments in behavioral studies when repeated measures are not feasible. Here, we found the boldness of brown trout to be positively correlated to δ^15^N; regardless of competition modes (sympatry or allopatry), fish length, or stream of origin. This indicates that bolder fish are feeding at a higher trophic level, as prey to consumer abundance of δ^15^N is typically a 3% increase (Jennings et al. 2001). Alternatively, the diet itself from higher trophic levels could make fish bolder. A similar effect to this has been found in male crickets ( *G. bimaculatus* ) where individuals feeding on high‐protein diets were more aggressive, active during mating, and behaviorally less stable than individuals fed on high‐carbohydrate diets (Han and Dingemanse 2017). A potential shortcoming in this study, however, is the small sample sizes that may affect the robustness of our conclusions, particularly when comparing different systems or behavioral categories. This should be borne in mind when evaluating our findings.

Our results, in part, support that a niche shift might be occurring due to the brook trout invasion. We also observed a correlation between the isotopic niche and personality of brown trout, which appears to be consistent across streams and competition modes. Interestingly, the competition between brown trout and invasive brook trout does not necessarily result in increased consumption of terrestrial prey, as suggested by some previous studies. This highlights the need for further research into the context dependency of this pattern. It could, for example, be speculated that the unexpected results could be due to a non‐linear response of brown trout to the increasing abundance and competition pressure from brook trout.

## Author Contributions

**B. Austad:** conceptualization (equal), data curation (equal), formal analysis (equal), visualization (supporting), writing – original draft (lead), writing – review and editing (lead). **L. Závorka:** conceptualization (equal), data curation (equal), formal analysis (lead), methodology (equal), project administration (equal), resources (supporting), software (equal), supervision (equal), validation (supporting), visualization (lead), writing – original draft (supporting), writing – review and editing (supporting). **J. Cucherousset:** conceptualization (supporting), methodology (equal), project administration (supporting), supervision (equal), writing – review and editing (equal). **J. Höjesjö:** conceptualization (equal), data curation (supporting), formal analysis (supporting), funding acquisition (lead), methodology (supporting), project administration (lead), resources (lead), supervision (lead), visualization (supporting), writing – original draft (supporting), writing – review and editing (equal).

## Conflicts of Interest

The authors declare no conflicts of interest.

## Supporting information


Appendix S1.

## Data Availability

The data supporting the findings of this study are available from the Dryad Digital Repository at the following link: http://datadryad.org/stash/share/ycBrSo8‐F‐w20jO80O4LatfVL6b3ai8yb01zfHtzeu0.
