# Supplementary material for: Effects of Brook Trout Invasion on Behavioral and Dietary Shifts in Brown Trout
Source: Ecol Evol. 2025 Mar 20;15(3):e70995. doi: 10.1002/ece3.70995 (PMC11925602; doi:10.1002/ece3.70995)
Supplement: Supplementary file 1 — Appendix S1. [file ECE3-15-e70995-s001.docx]

**Appendix**

*Appendix A: Supplementary Tables*

**Table A1***: Fork length and number of fish sampled from each species and stream stretch, used in boldness scoring.*

| Stream section (abbreviation) | Brook trout: FL±SD mm (N) | Brown trout: FL±SD mm (N) |
| --- | --- | --- |
| Ringsbäcken allopatry (RA) | -- | 135 ± 28 (51) |
| Ringsbäcken sympatry (RS) | 137 ± 23 (25) | 143± 32 (24) |
| Lindåsabäcken allopatry (LA) | -- | 145 ± 22 (41) |
| Lindåsabäcken sympatry (LS) | 138 ± 21 (35) | 129 ± 25 (9) |

**Table A2**: *Stable isotope values (δ^13^C and δ^15^N) of each consumer group without correction for the prey baseline, and number of sampled individuals. All fish come from the same groups summarized in Table A1.*

| Stream section | δ^13^C±SD ‰: Brown | δ^15^N±SD‰: Brown | N | δ^13^C±SD‰: Brook | δ^15^N±SD‰: Brook | N |
| --- | --- | --- | --- | --- | --- | --- |
| RA | -26.6±1.1 | 10.1±0.8 | 14 | -- | -- | -- |
| RS | -26.6±0.4 | 8.7±0.6 | 8 | -26.7±0.4 | 8.4±0.4 | 7 |
| LA | -26.6±0.7 | 8.6±0.5 | 14 | -- | -- | -- |
| LS | -26.6±0.9 | 9.9±0.4 | 7 | -26.2±0.3 | 9.9±0.4 | 8 |

**Table A3**: *Prey items collected from each stream site. Samples were identified to family/taxa and sorted (all samples specific to where they were captured) and used in stable isotope analysis.*

| **Stream section** | **Habitat** | **Order/Class** | **Family/Taxa** |
| --- | --- | --- | --- |
| LA | Aquatic | Diptera | Athericidae (Atherix sp.) |
| LA | Aquatic | Ephemeroptera | Ephemeridae (Ephemera sp.) |
| LA | Aquatic | Ephemeroptera | Baetidae (Baetis sp.) |
| LA | Aquatic | Ephemeroptera | Heptageniidae (Heptagenia sp.) |
| LA | Aquatic | Gastropoda | Gastropoda |
| LA | Aquatic | Trichoptera | Polycentropodidae |
| LA | Aquatic | Trichoptera | Hydropsychidae (Hydropsyche sp.) |
| LA | Aquatic | Trichoptera | Polycentropodidae |
| LA | Terrestrial | Araneae | Araneae |
| LA | Terrestrial | Coleoptera | Curculionidae |
| LA | Terrestrial | Collembola | Collembola |
| LA | Terrestrial | Dermaptera | Dermaptera |
| LS | Aquatic | Coleoptera | Elmidae (Limnus sp.) |
| LS | Aquatic | Diptera | Athericidae (Atherix sp.) |
| LS | Aquatic | Ephemeroptera | Heptageniidae (Heptagenia sp.) |
| LS | Aquatic | Oligochaeta | Oligochaeta |
| LS | Aquatic | Plecoptera | Leuctridae (Leuctra sp.) |
| LS | Aquatic | Trichoptera | Hydropsychidae (Hydropsyche sp.) |
| LS | Aquatic | Trichoptera | Polycentropodidae |
| LS | Terrestrial | Araneae | Araneae |
| LS | Terrestrial | Coleoptera | Curculionidae |
| LS | Terrestrial | Collembola | Collembola |
| LS | Terrestrial | Hemiptera | Hemiptera |
| LS | Terrestrial | Hymenoptera | Hymenoptera |
| LS | Terrestrial | Hymenoptera | Formicidae (Formica sp.) |
| LS | Terrestrial | Hymenoptera | Formicidae (Formica sp.) |
| LS | Terrestrial | Opiliones | Opiliones |
| RA | Aquatic | Coleoptera | Elmidae (Limnus sp.) |
| RA | Aquatic | Ephemeroptera | Ephemeridae (Ephemera sp.) |
| RA | Aquatic | Ephemeroptera | Heptageniidae (Heptagenia sp.) |
| RA | Aquatic | Odonata | Odonata |
| RA | Aquatic | Plecoptera | Leuctridae (Leuctra sp.) |
| RA | Aquatic | Trichoptera | Polycentropodidae |
| RA | Terrestrial | Araneae | Araneae |
| RA | Terrestrial | Araneae | Araneae |
| RA | Terrestrial | Coleoptera | Curculionidae |
| RA | Terrestrial | Hemiptera | Miridae |
| RA | Terrestrial | Hemiptera | Hemiptera |
| RA | Terrestrial | Hymenoptera | Formicidae (Formica sp.) |
| RA | Terrestrial | Opiliones | Opiliones |
| RS | Aquatic | Amphipoda | Gammaridae (Gammarus sp.) |
| RS | Aquatic | Isopoda | Asellidae (Asellus sp.) |
| RS | Aquatic | Trichoptera | Rhyacophilidae (Rhyacophila sp.) |
| RS | Aquatic | Trichoptera | Polycentropodidae |
| RS | Aquatic | Trichoptera | Polycentropodidae |
| RS | Aquatic | Trichoptera | Hydropsychidae (Hydropsyche sp.) |
| RS | Terrestrial | Araneae | Araneae |
| RS | Terrestrial | Araneae | Araneae |
| RS | Terrestrial | Coleoptera | Curculionidae |
| RS | Terrestrial | Collembola | Collembola |
| RS | Terrestrial | Hemiptera | Miridae |
| RS | Terrestrial | Hymenoptera | Hymenoptera |
| RS | Terrestrial | Hymenoptera | Formicidae (Formica sp.) |
